# Evaluating the effectiveness of hazard mapping as climate change adaptation for community planning in degrading permafrost terrain

**DOI:** 10.1007/s11625-018-0614-x

**Published:** 2018-08-04

**Authors:** Melanie Flynn, James D. Ford, Jolène Labbé, Lothar Schrott, Shirley Tagalik

**Affiliations:** 1 Priestley International Centre for Climate, University of Leeds, Leeds, UK; 2 McGill University, Montreal, Canada; 3 University of Bonn, United Nations University-Environmental Risk and Human Security, Bonn, Germany; 4 The Arviat Wellness Centre/Aqqiumavvik Society, Arviat, Canada

**Keywords:** Climate change, Permafrost degradation, Hazard mapping, Adaptation evaluation

## Abstract

Permafrost in northern Canada is susceptible to degradation due to rapid climate change, with hazard mapping promoted as an important activity to guide sustainable community adaptation and planning. This paper presents a framework for evaluating permafrost mapping exercises designed to inform climate change adaptation actions. We apply the framework using a case study of the Incorporating Climate Change into Land Development—Terrain Analysis project (ICCiLD). ICCiLD is a hazard mapping project utilizing interferometric synthetic aperture radar to monitor ground disturbance and categorize land development suitability in seven communities in the territory of Nunavut, Canada. We looked at one of the communities, Arviat, as our case study. We examined technical data and drew upon semi-structured interviews (*n* = 19) with map creators and users. We found ICCiLD added new and relevant information for community planning, increased awareness of the risks posed by permafrost thaw and built stakeholder relations. Strong coordination and high public consciousness of local climate impacts emerged as key factors underpinning project success. Nevertheless, in the case of Arviat, the effectiveness of the hazard maps in influencing land-use planning was constrained by communication challenges between project creators and end-users. These challenges included limited community access to the data and uncertainty surrounding how to operationalize the map suitability classifications. Broader climate change adaptation challenges included the presence of other more immediate community planning priorities and a limited ability to incorporate Indigenous ways of knowing into a technical mapping project. The lessons from this evaluation provide insight for the development of mapping-based adaptations across Arctic regions.

**Electronic supplementary material:**

The online version of this article (10.1007/s11625-018-0614-x) contains supplementary material, which is available to authorized users.

## Introduction

Permafrost is the ground that remains at or below 0 °C for at least 2 consecutive years. It covers approximately 24% of the earth’s surface (Larsen and Anisimov 2014). Air temperature and hydrological cycles are major determinants of permafrost occurrence and climate change is expected to have significant impacts on permafrost degradation, with polar regions projected to experience the greatest warming of any global region this century (Larsen and Anisimov 2014). Nearly 50% of permafrost in Canada is at high or moderate risk to permafrost thaw resulting from warming temperatures (Smith and Burgess 2004). In addition to rising temperatures, a circumpolar reduction in snow cover of between 7 and 25% will adversely impact permafrost, resulting in a decrease between 37 and 81% of near-surface permafrost by the end of the century (Collins et al. 2013).

Climate change-induced impacts on permafrost include, increasing ground temperature, thawing ground ice within permafrost, and increased active layer thickness, all of which can contribute to the thinning and decreased aerial extent of permafrost. Permafrost degradation, in turn, has several potential primary impacts on the physical environment, including enhanced slope instability, accelerated coastal erosion, amplification of surface warming due to enhanced CO_2_ and CH_4_ emissions, altered ecosystems through changes in hydrological systems, and surface subsidence (Lamoureux et al. 2015; Romanovsky et al. 2002; Vaughan 2013). Secondary impacts include infrastructural instability caused by increased erosion and surface subsidence. In the Canadian Arctic, for example, transport systems are affected by permafrost degradation on airport runways (Hawkins 2013) and highways (Calmels et al. 2015a, b). The viability of some communities is being challenged by coastal erosion exacerbated by permafrost degradation (Bronen 2010; Ford et al. 2015). Additionally, traditional activities are affected by melting ice cellars used to store locally harvested meat (Lamoureux et al. 2015; Nyland 2016; Shiklomanov 2016) and through the reduction of access to and availability of traditional country food (Calmels et al. 2015a, b).

In response to these experienced and projected impacts, communities and decision makers are identifying opportunities for adaptation to manage the impacts of permafrost degradation on infrastructure and livelihoods (Ford et al. 2015). The Intergovernmental Panel on Climate Change (IPCC) describes adaptation as “the process of adjustment to actual or expected climate and its effects, in order to either lessen or avoid harm or exploit beneficial opportunities” (IPCC 2014). Numerous adaptations are occurring in response to permafrost degradation. Efforts to protect building stability, for instance, include retrofitting older infrastructure through drainage improvement and removal of skirting around buildings to improve airflow (Calmels et al. 2016). Additionally, new infrastructure adaptation includes the utilization of adaptive foundation types such as spaceframes to minimize heat transfer and maximize ground cooling (Lamoureux et al. 2015). In some regions of the Russian Arctic, adaptation is occurring through the demolition of unstable buildings (leaving the foundation structures in place) allowing the ground to refreeze and then rebuilding these structures using lighter building materials (Nyland 2016; Shiklomanov 2016). In other cases, thermosyphons are installed, using heat transfer fluid to remove heat from foundations of infrastructure projects (Lamoureux et al. 2015; McGregor et al. 2008). Adaptation of key transportation infrastructure in the northern regions includes the recent extensive monitoring of permafrost at Iqaluit Airport to determine ideal location for the building of a new runway (Mathon-Dufour et al. 2015) and the testing of new culvert drainage systems on the Alaska Highway to minimize permafrost thaw (Transport Canada 2015).

In addition to technologies aimed at mitigating permafrost thaw impacts on existing infrastructure, the utilization of hazard mapping for monitoring and categorizing areas of risk to identify preferential areas for development is the key to adaptation in permafrost environments (Champalle et al. 2013). Planning for and making infrastructure decisions based on potential permafrost thaw in Northern communities is more cost-effective adaptation than retrofitting infrastructure, (Melvin et al. 2017) and development suitability and hazard mapping have a large part to play in supporting local planning decisions. Hazard mapping is a spatial representation of risk associated with a specified hazard (Champalle et al. 2013) and can determine the current and future hazard risk to an area (Preston et al. 2011). Hazard mapping has a long tradition in disaster risk reduction and risk communication (Preston et al. 2011). It is also used for climate adaptation applications in northern Canada for risks associated with permafrost, coastal hazards, landslides, sea ice, riverine flooding and forest fires (Champalle et al. 2013; Collaborative for Advanced Landscape Planning 2016; Hatcher et al. 2011; Sheppard 2012). There has been widespread use of hazard mapping across the northern regions of Canada to prepare and plan for climate change in a region which is expected to be significantly impacted by warming temperatures. Champalle et al. (2013) provide an extensive review of hazard mapping utilization for adaptation in the built environment in northern Canada and identify barriers impeding utilization of hazard maps. The barriers include, a limited end-user awareness of the existence of these maps, coupled with a mismatch in the way that data are made available meaning end-users are unable to access and view hazard maps. The review also suggests that a closer working relationship between mapping experts and end-users would be beneficial in increasing operability, understanding, and trust in outputs. Despite increasing interest in hazard mapping, few studies have evaluated how such maps are used in decision-making or documented the perspectives of end-users (Ford et al. 2018; Preston et al. 2011). This is a missed opportunity for assessing the effectiveness of hazards maps, learning what works and what does not, and for sharing good practices and experiences (Bours et al. 2014b, 2015; Ford and Berrang Ford 2015).

This paper presents a framework for evaluating mapping projects which seek to inform adaptation decision-making. We apply the framework using a case study of Arviat, Nunavut and the Incorporating Climate Change into Land Development—Terrain Analysis project (referred to herein as “ICCiLD”). ICCiLD is a mapping project utilizing interferometric synthetic aperture radar (InSAR) to monitor ground disturbance and categorize development suitability in communities underlain by permafrost. Though the ICCiLD project utilized InSAR data to create the maps, there are multiple approaches for creating hazard maps. This evaluation will provide some insight into the use of the InSAR technique whilst also considering the applicability and usability of mapping-based adaptations more broadly across Arctic regions.

## Study area

### Study site: Arviat, Nunavut

**Fig. 1:**
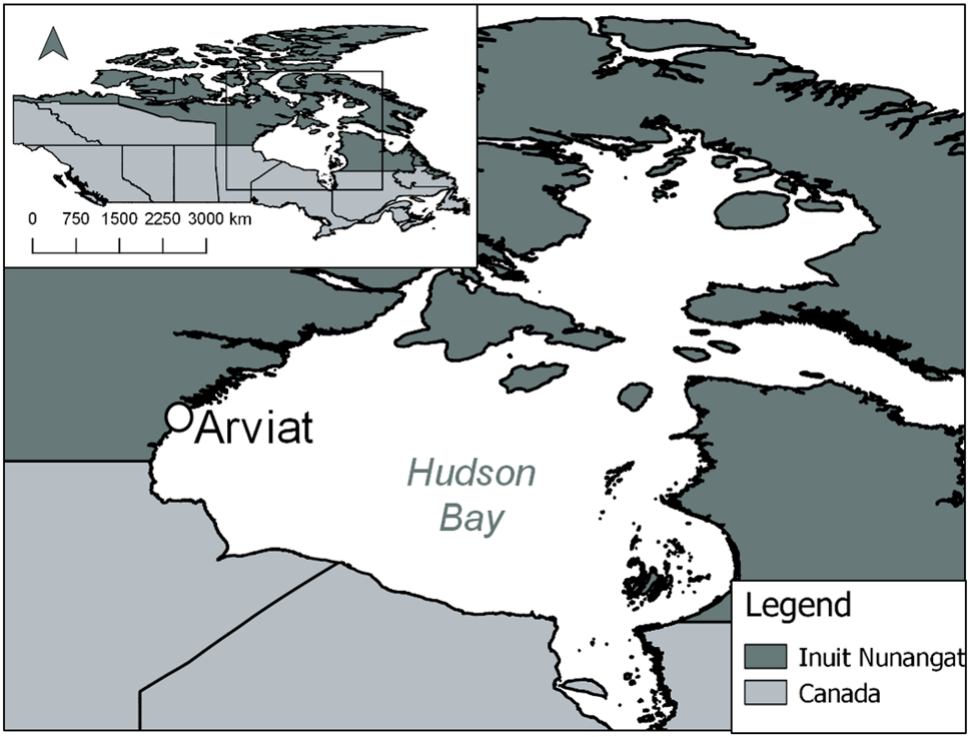
Location of our study site, community of Arviat, Nunavut, Canada. Map produced by authors with data from Indigenous and Northern Affairs Canada (2017) and Statistics Canada (2016)

Our evaluation focuses on the community of Arviat, Nunavut. Nunavut is the newest territory in Canada, established in 1999 by the Nunavut Land Claims Agreement (Government of Nunavut 2014) (population: 37,315, 84% Inuit). The territory is spread across 1.9 million km^2^ and contains 26 small communities, all of which are accessible only by plane or by boat in summer months (Government of Nunavut 2016; Nunavut Bureau of Statistics 2015). Nunavut has warmed by between 1.6 and 2.6 °C since the 1960s (Environment Canada, 2015), permafrost temperature across the territory has seen a warming of between 0.04 and 0.25 °C per year since 2008 (Ednie and Smith 2015). The community of Arviat (population: 2514, 94% Inuit), is located on the west coast of Hudson Bay (Fig. 1). Arviat is situated on continuous permafrost, and existing infrastructure is exhibiting signs of impact as a result of permafrost thaw, including frost jacking, thaw settlement and possible instability related to saline permafrost (Forbes et al. 2014). Importantly, the community is growing rapidly, with a projected increase in population of > 60% in the next 20 years (Nunavut Bureau of Statistics 2015) and there is a high demand for new housing (Forbes et al. 2014). Arviat completed hazard mapping to help identify suitable areas for future development and was selected for this evaluation because the community was a major hub for the ICCiLD project and received additional community outreach work.

### The Incorporating Climate Change into Land Development—Terrain Analysis project

ICCiLD was a 4-year project (2012–2016) funded by Aboriginal Affairs and Northern Development Canada (AANDC)^1^ and undertaken in seven communities in Nunavut. ICCiLD was created to assist Government of Nunavut community planners and the selected communities in identifying suitable ground for future community development through mapping areas of thaw-sensitive permafrost. The project created permafrost vulnerability maps (also known as hazards maps) using Interferometry Satellite Aperture Radar (InSAR) data obtained from RADARSAT-2 to measure surface deformation at regular intervals (24-day cycle). Terrain elevation changes were overlain throughout several imaging periods to determine changes over time; these data are used to indicate permafrost processes, such as thaw settlement or subsidence (Kääb 2008). These data were used to create development suitability maps where geospatial data concerning a hazard (in this case, permafrost thaw) are utilized to categorize the suitability of the area for development. ICCiLD focused on communities with a limited presence of bedrock (where building foundations were more likely to be impacted by permafrost thaw), and where previous satellite monitoring had occurred to monitor change over time (3vGeomatics Inc. and BCG Engineering Inc. 2011). In addition, the project developers also prioritized communities where substantial future development was expected to occur.

ICCiLD adopted several techniques to manage uncertainties and difficulties of data collection. In the case of Arviat, the substantial number of lakes and intertidal areas resulted in low coherence in data, as these areas change more over time, known as temporal decorrelation (3vGeomatics Inc. 2013). This low coherence meant that additional scenes (*n* = 34) were collected to allow Arviat’s data stack to reach ‘maturity’, the stage where additional processing techniques can be applied to the data to improve accuracy (3vGeomatics Inc. 2015). The high degree of standing water in Arviat during summer months made it difficult to determine true ground movement, as a result, better quality interferograms^2^ were often obtained during winter months when waterbodies were frozen (3vGeomatics Inc. 2015). Despite these issues with data quality, according to interviewees, the map for Arviat corroborated with the surficial geology data collection in Arviat which is currently being processed by Memorial University (Bagnall 2014).

The map suitability criteria were based on InSAR data and geospatial characteristics and were defined in 3vGeomatics Inc. (2013) as:

- *Suitable for development* The area is thought to be stable and available data indicate little or no evidence of ice-rich and changing permafrost conditions. Additional characteristics of the area include exposed rock, bare soil, low vegetation, a slope < 4%, or a non-south facing aspect.
- *Possibly suitable for development* The area is possibly stable for development as ground conditions have limited indicators of changing permafrost conditions. In some cases, due to the lack of quality remote sensing data, the presence of permafrost could not be ruled out. Additional characteristics of the area include exposed rock, bare soil, low vegetation, a slope < 4%, or a non-south facing aspect.
- *Marginally suitable for development* All data indicate that some ground ice is present, and the area is, therefore, only marginally suitable for future development. Additional characteristics of the area include low vegetation or a slope 4°–10° (includes south facing).
- *Unsuitable for development* An area of rugged terrain, evidence of ground ice or subsidence, or surface water identified in the area. Additional characteristics of the area include wet areas within 25 m of displacement, those within 30 m of a water body,^3^ or slopes > 10%.

**Fig. 2:**
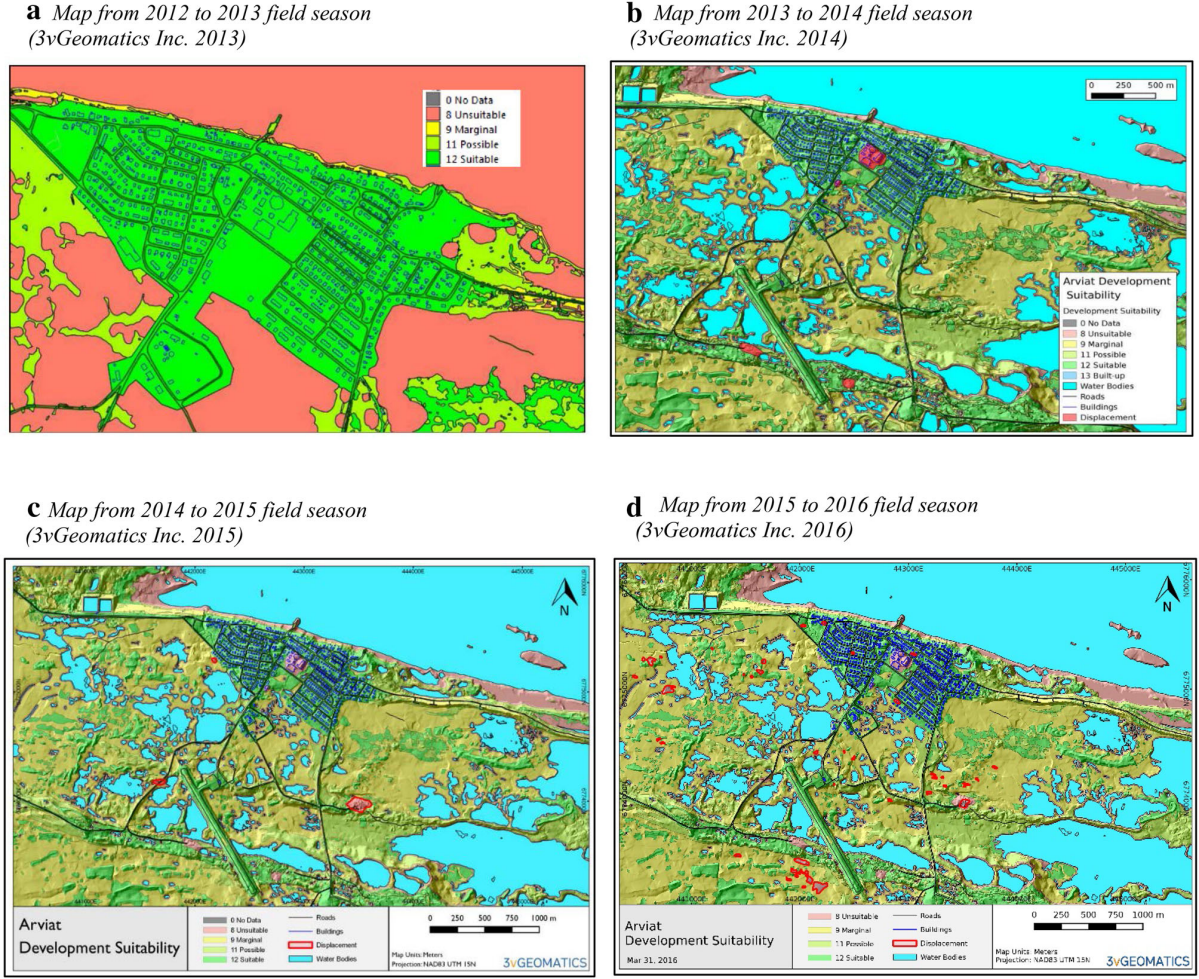
Development suitability maps for Arviat

The ICCiLD map was updated annually over 4 years (2012–2016). Multi-year data allowed for additional processing to model active layer thickness, allowing for a greater separation of annual cyclical ground movement from long-term ground movement (3vGeomatics Inc. 2015) and the improved identification of smaller pockets of displacement (3vGeomatics Inc. 2016). Each of the updated maps for Arviat (*n* = 4) demonstrated an increased level of complexity (see Fig. 2). Nevertheless, local ground conditions in Arviat limited the overall confidence level^4^ of the map produced for Arviat to ‘moderate’.

The need for additional engagement was identified during the third year of ICCiLD by the project creators. The engagement was designed to share the preliminary maps and discuss incorporating climate change adaptation in community infrastructure planning. Arviat was chosen for this engagement activity due to strong community support for the project, the demand for new housing units, the initial mapping results showing Arviat’s ice-rich permafrost, and because the Nunavut Housing Corporation’s head office is located there. Arviat also received additional in situ testing performed by students from Memorial University including the sampling of soil in and around Arviat.

## Methodology

### Evaluation framework

A framework to structure the evaluation of the ICCiLD project was developed,^5^ drawing upon the general adaptation evaluation scholarship and work on hazard mapping. The framework comprised a three-step process which included: (1) providing a baseline evaluation, (2) defining and evaluating success and (3) characterizing the adaptive environment (see evaluation framework in Fig. 1 supplementary material).

#### Baseline characterization

When evaluating the impact of hazard mapping projects for informing adaptation decision-making, it is important to establish a knowledge baseline prior to the project’s initiation (Gunson 2015). This baseline identifies how much additional information the project created, the extent to which pertinent gaps in understanding were addressed, and any remaining knowledge gaps. A review of the peer-reviewed and grey literature (*n* = 17 documents) was conducted to establish the baseline, focusing on studies published over the last two decades. Semi-structured interviews were also conducted with Government of Nunavut employees (*n* = 4) and housing and infrastructure specialists in Arviat (*n* = 6), to determine what data they were using in their decision-making prior to the creation of the ICCiLD map.

#### Defining and evaluating success

Defining success in adaptation projects is challenging, as what comprises ‘good adaptation’ and thus ‘good hazard maps’ is highly subjective and contextual (Bours et al. 2014a, b; Wise et al. 2014). This is particularly pertinent in Nunavut, where Inuit worldviews and cultural practices shape the context within which science is conducted and where community members retain a significant understanding of the natural environment (Labbé et al. 2017). Consequently, the second step in the evaluation framework created a logic model to define successful adaptation in the context of ICCiLD. The model highlights connections between inputs, activities and outputs; considers the appropriateness of project assumptions; documents stakeholder expectations; and examines why some outcomes may or may not have occurred (AUSAID 2005; Krause et al. 2015; UKCIP 2013). The logic model created to evaluate ICCiLD contained five components:

- *Inputs* The resources required to undertake the work and produce the desired project outputs (e.g. staffing and financial resources).
- *Activities* The tasks to be undertaken as part of the project delivery (e.g. collect raw data).
- *Outputs* The tangible products produced during the project (e.g. hazard maps).
- *Outcome* The desired achievements expected from the project (e.g. increased knowledge of local climatic impacts).
- *Impact* The macro-level objectives to which this project is designed to contribute (e.g. reduced vulnerability to permafrost degradation).

The logic model was created by extracting information on the key components of the project based on information from the ICCiLD project funding proposal and the project’s annual reports. We then verified this information with key stakeholders. By outlining the key components involved in project success, we identified the key assumptions that may impact the success of the project (e.g. key stakeholders will understand and use the hazard maps produced). These assumptions were used in the construction of the evaluation interview questions (see data collection, Sect. 3.2): for example, “Is this map easy to understand?” and “Were all relevant stakeholders present at meetings? If not, who was missing?”

Once the key components of success in the context of the ICCiLD project were defined, we aimed to provide an overview of current project performance and processes to create project-specific feedback on performance including, data collection methods, stakeholder engagement, and map usability and access. The information was based on semi-structured interviews (*n* = 19) and a critical analysis of the maps produced.

#### Characterizing the adaptive environment

The current and future success and influence of an adaptation project is determined not only by the technical aspects of the work, but also by how the project leverages and links to the broader institutional environment which may support or constrain adaptation (Ford and King 2015; Measham et al. 2011; Moser and Ekstrom 2010). Therefore, we examined the likelihood that the ICCiLD project will inform decision-making on adaptation to permafrost change in the short-, medium-, and long-term (i.e. inform land-use planning through the utilization of the suitability maps in community planning documents). We applied a modified version of Ford and King’s (2015) and Ford et al. (2017) adaptation readiness framework, considering how the following factors act as barriers or enablers to project success:

- *Funding and resources* The inputs made available to facilitate adaptation and are important for ensuring that the output of an adaptation project is supported on completion (e.g. resources are made available for utilizing hazard maps in land-use planning). Many adaptation projects identify opportunities for adaptation which remain unrealized due to an absence of resources for implementation (Champalle et al. 2015).
- *Institutional organization* The role of organizations (e.g. government departments, community bodies) in coordinating and directing adaptation activities. In absence of such coordination, adaptation projects are often ad hoc and lack the strategic guidance necessary for effective implementation (Labbé et al. 2017; Smith et al. 2009).
- *Usable science* The extent to which the project works closely with knowledge users to ensure the work is pertinent, timely and integrates necessary information to inform decisions (Ford et al. 2013). Failure to do this can result in research that is not trusted, understood, or is supplied too late (or early) in decision cycles (Ford and King 2015; Lemos 2015; Lemos and Morehouse 2005).
- *Decision*-*making* The way in which key decision makers are engaged in adaptation projects. Decision makers must act in situations of climatic uncertainty with competing needs and priorities and this should be built into project design (Ford and King 2015; Henstra 2015).
- *Political leadership* The notion that for adaptation projects to influence decision-making, it requires support and leadership from high levels to ensure adaptation is integrated into ongoing planning (Henstra 2015).
- *Public support* The degree of understanding and backing from the general population for acting on climate change, particularly in cases where adaptation involves financial costs or disproportionately affects certain households or communities. This is influenced by risk perception and past experiences of climate change impacts (García de Jalón et al. 2013; Vignola et al. 2012).
- *Stakeholder engagement* Which stakeholders were involved in the process, how they can influence adaptation and what stages of the process they were involved in? For example, those stakeholders involved at the initial stages of the project may be able to influence the project design. Stakeholder engagement is recognized to improve the effectiveness of adaptation implementation (Sherman and Ford 2014).
- *Indigenous knowledge* In the case of Nunavut, we use the term *Inuit Qaujimajatuqangit* (*IQ*)*. IQ* is a collection of societal values held and adhered to by Inuit (e.g. the value *Tunnganarniq*, which means fostering good spirits by being open, welcoming and inclusive) (Government of Nunavut 2015). Since the creation of Nunavut in 1999, policy making in the territory is mandated to incorporate Inuit Societal Values held and adhered to by Inuit communities (Government of Nunavut 2015; NTI et al. 2010). These values are integrated in adaptation decision-making in the territory and as a result are an important factor to consider (Labbé et al. 2017).

### Data collection

Nineteen semi-structured interviews were conducted (15 face-to-face interviews, 4 phone interviews) to document perspectives on the usability of the maps and information produced. Purposive sampling was used, and participants were selected based on their roles within the ICCiLD project. We identified five different interviewee types: creators (technical), creators (community outreach), users (technical), users (community outreach), and users (mixed) (see interview categories in supplementary materials, Table 1). Interviews lasted between 15 min and 1.5 h and an Inuktitut interpreter was used when required. The semi-structured interview guides were divided into the four sub-components: (1) what problem is the project trying to address? (2) What is the project supposed to do? (3) How did the project do? (4) How does that fit into the big picture? During the interview, the most recent version of the map available at that time (2014/2015 version) was shown to interviewees.

**Table 1:** Adaptation readiness factors and examples Modified from Ford and King (2015)

Readiness factor	Indicator	Example
Funding and resources	Dedicated funding streams or budgets available within departments for climate change adaptation work (Ford and King 2015)	Organizations interviewed currently found money for adaptation from other budgets (e.g. Halloween indoor activities held by Arviat). Department of Environment—Climate Change Section works with GN Departments to find federal funding opportunities for climate change adaptation projects
	Climate change adaptation funding is being accessed and utilized	Community and Government Services were able to access Aboriginal Affairs and Northern Development Canada funds Arviat Wellness Centre/Aqqiumavvik Society also accessed funds from national level. They did not provide funding for this project, but did provide time in-kind. The center has previously received climate change funding for other projects Laval and Memorial’s work was funded nationally through ArcticNet
Institutional organization	Presence of boundary organizations working on climate change adaptation (Ford and King 2015)	The bringing in of Department of Environment—Climate Change Section to work with project leader and coordinate outreach
	Stakeholders were involved in the decision-making process (Ford and King 2015)	Climate change engagement in Arviat brought together end-users with map creators to discuss results and next steps
Usable science	Quality, timeliness and pertinence (Ford et al. 2013)	*Quality* Literature review showed the project is using appropriate technology and in situ data to validate results *Timeliness* Community and Government Services felt project outputs would be ready for incorporation in official community plan. Some felt it was too late for current development occurring in unsuitable zones *Pertinence* The project provided new knowledge, but the suitability categories were critiqued
	Meaningful consultation with end-users	3vGeomatics consulted with Community and Government Services (end-user). However, the Hamlet of Arviat were not consulted during project creation and did not have significant input prior to community engagement
Decision-making	Access to key project information for decision makers	Community and Government Services and the Hamlet of Arviat had access to maps, but not all potential users had access to the map or knew where to find the information
	Climate change adaptation is considered and accounted for in decisions made	Other development priorities were given more consideration than climate change in development decisions (e.g. cost, desirability of location, quality of life)
Leadership	Organizations or departments are mandated to include climate change in their work	Most organizations did not have climate change policies, except for the GN’s CCS. There are other northern climate change standards and policies that the GN are encouraged to follow, but have been developed by other organizations
	Statements of importance and need for adaptation by leaders (Ford and King 2015)	Community and Government Services felt it would be irresponsible to not include climate change considerations into community planning. The Hamlet of Arviat felt there was too much uncertainty in impacts and Arviat currently had greater needs than adaptation (e.g. housing crisis)
Public support	There is a public perception of the importance of climate change adaptation (Ford and King 2015)	40–50 people attended the public event held, interviewees acknowledged changes happening in Arviat and discussed adaptation
	Public understanding of climate change and impacts	Unpredictability of weather and changing migration patterns were discussed by interviewees. However, some misconceptions about the link between impacts and climate change exist
Stakeholder engagement	Relevant stakeholders have been engaged	Interviewees agreed that key stakeholders were present during the outreach
	Stakeholders understood how this project would be utilized in their day-to-day role	Key stakeholders did not feel that they were primarily responsible for development decisions in Arviat. As a result, they felt that the map was not linked to decisions which they were able to influence
Indigenous knowledge—Inuit Qaujimajatuqangit (IQ)	IQ was collected during the project	Discussions with Elders occurred and local knowledge was sought out through field visits, ArcticNet work and community engagement
	IQ is integrated into project	Summary documents of Elder’s meetings were shared with stakeholders, including map creators, but the information has not been added as an additional layer into the maps. Interviewees discussed the difficulty in incorporating IQ

Authors have modified this framework providing an additional readiness factor (Indigenous knowledge) and through the provision of new indicators. All examples provided are author’s own to fit context of this study

### Analysis

Interviews were transcribed and then coded based on two approaches proposed by Auerbach and Silverstein (2003). First, magnitude coding was used to extract project-specific feedback on ICCiLD, whereby statements relating to the maps produced were coded as positive (+), negative (−), or as a statement recommending improvement (REC). A total of 479 comments were documented and coded in this way (218 positives, 143 negatives, and 118 recommendations). Secondly, elaborative coding was used to examine the adaptation readiness factors (outlined in Table 1), identifying multi-scalar linkages, and enablers and barriers for adaptation decision-making. For example, through determining public understanding and perception of climate change, we considered how public support was likely to affect the adaptation process in Arviat. Additionally, by identifying key decision makers in the field of community development and discussing their perceived needs and priorities, we determined if and how climate change will be incorporated into local housing planning.

### Methodological limitations

The long timescales of climate change and the uncertainty of feedback systems mean that adaptation can be considered as a process of continual adjustment (Bours et al. 2014b; Ford et al. 2018). This makes defining a successful endpoint for adaptation challenging. Consequently, this paper considers adaptation as a continually evolving process and chooses to evaluate the processes or strategies of adaptation rather than the specific outcomes. In practice, this means that this paper applied a process-based evaluation, exploring the perceived usability of the categories identified in the map, the outreach and engagement methods used during the project and the local interpretation of the quality and usability of these maps.

## Results

### The potential of the ICCiLD project to influence community development

The ICCiLD is on-track to achieve the overall project outcomes (see logic model in Fig. 2 supplementary material). Positive feedback comprised two main themes. First, the project improved locally relevant information for decision-making including: local agreement with the 2014/2015 version of the map (*n* = 22), relevant information for local decision-making (*n* = 18), and consideration of the local context of the area in map creation (*n* = 16). Many of the users interviewed (*n* = 18) believed that this map would provide helpful information for making decisions on future development projects. Participant comments include:

If this data […] was made available to us and there were high-risk areas where we had units, we would certainly develop plans around that. (Project user). The idea is to incorporate these maps and this research into future community plans and zoning bylaws, […] steering future growth into areas that are more suitable and have less issues with permafrost melting and huge changes in elevation and grade. (Project creator).

Second, interviewees noted that the project increased communication and relationship building including: improved local knowledge sharing (*n* = 22), increased relationship building (*n* = 16), and aided dissemination of results (*n* = 11). The logic model (Fig. 2 supplementary material) documented some unexpected inputs, activities and outcomes, due to additional community engagement activities added during the ICCILD project. These additional activities provided added value, particularly in building synergy with ongoing research, and encouraging communication and collaboration. To date, two of the seven ICCiLD communities (Arviat and Cape Dorset) have received additional community engagement. The ICCiLD project’s community engagement activities connected key stakeholders from the government, research and community level. This connection facilitated communication of results, the collaboration between groups, and created a cohesive narrative for permafrost research. Within the project, stakeholder meetings were well-attended and key end-users such as Hamlet, community planning and infrastructure staff at the territorial level, and staff from the Arviat Housing Authority participated. One interview participant commented:

I think we tried to include everybody […] the focus was on community and community infrastructure and so they focused on housing, trades and construction people, local businesses and then, the Hamlet (Project creator).

Although the primary focus of the engagement sessions was to share the hazard map and discuss *where* to build, there were also interesting conversations around *how* to build infrastructure, including choosing the right foundation type. Interviewees expressed that there are still gaps in decision-making between those who choose building site locations and those who build infrastructure. Those responsible for building housing felt that their needs were not captured in the map, as this interviewee outlines:

Just due to the nature of how construction works in Nunavut from [our] standpoint we largely build where we’re told to build (Project user).

This quote highlights the complexity of infrastructure development and the need for better coordination between all players.

Negative interviewee feedback included: limited access to data (*n* = 14), contradictory local knowledge of development suitability areas (*n* = 13), the poor timing of the delivery of project information (*n* = 6), and a lack of understanding regarding the suitability categories in the maps produced (*n* = 5). Interviewees commented on limited access to data during our evaluation (summer 2015). Though key project creators, for example, the Hamlet, the Government of Nunavut Community and Government Services and the Department of Environment—Climate Change Section, had access to maps throughout the process. Other key community stakeholders, including the Nunavut Housing Corporation and the local Housing Authority, reported not being able to access or use the maps. Consequently, there was no opportunity for them to use this information in development decisions during the first 3 years of the project. The maps have since been uploaded to the Nunavut Climate Change Centre website, along with a plain language summary (http://climatechangenunavut.ca/), thus potentially increasing accessibility to a broader array of users and encouraging community members to take a more active interest in development in Arviat.

Interviewees raised concerns regarding contradictory local knowledge of development suitability areas as compared with suitability indices created using the map data. Generally, local people agreed with most of the areas highlighted as suitable or unsuitable on the map, noting that the eskers in the community provided the most stable ground. Despite this, there was an area ranked as suitable on the hazard map which several residents felt was not suitable for building on. The ground under this spot was described as “alluvial sediment”, “quick sand” and “cement” by four interviewees, all of whom were Elders or long-term community members. Those community members believed houses built in this area would “slide down”. Conflicting knowledge resulted in some community members expressing distrust in map accuracy.

The timeliness of the project was met with mixed opinions. The information was timely in relation to the integration of the map into the official community plan for Arviat (a key project outcome). Conversely, some local stakeholders felt that providing a map which shows areas categorized as ‘unsuitable’ or ‘marginal’ in places where current development is occurring, or future development is planned was inappropriate:

They said, [you have] come with this information way too late, we’re developing 5 and 10-year plans and so we’re building in all those sites, the decision [has] been made, can’t be undone (Project creator).

Other interviewees disagreed; however, believing the maps added information for those responsible for building maintenance lifecycle planning:

We develop long-term maintenance cycle plans for all our units so we’d definitely have that in there (Project user).

A lack of understanding regarding the suitability categories in the maps produced was also documented and for some interviewees, the categories lacked the clarity required for utilization. There is no guide provided with the map outlining how categories were determined, with the guide categories of ‘possible’ and ‘marginal’ providing unclear boundary information for building site selection. This is exemplified by one informant’s comment:

I mean, possible/suitable for what? Are some sites suitable for single family dwelling but may not be suitable for a larger multiplex building. Because something is unsuitable or marginal we’re just not going to put anything there? (Project user).

To make this information operational at the construction level, some interviewees felt that an accompanying user guide was necessary. There has since been a legend developed to give more information on the suitability categories.

Interviewees provided recommendations for improving the usability of the maps produced. Recommendations included adding more engaging or oral activities (*n* = 6). ICCiLD users commented that knowledge sharing at the community level was good (*n* = 22) and that using more of these engagement activities in the future was advisable. Youth were engaged in filming permafrost monitoring and community demonstrations of research equipment were given. Additionally, the installation of a community permafrost monitoring site in Arviat was mentioned by members of the community. Training on how to obtain the data from the site was forthcoming and the community had plans to extract and use the data. When interviewed, creators believed that if appropriate people were identified in the community, minimal training would be required to engage those interested in making on-the-ground observations. This would allow additional skill sharing at the community level, save money, and improve the accuracy of project mapping data. Other recommendations included ‘consider the local quality of life’ (*n* = 4) and ‘clarify the ranking system used’ (*n* = 4). Finally, select users made a general recommendation concerning development in Arviat (e.g. ‘do not build on water’, *n* = 5). This suggestion implies community members are concerned with community drainage issues, interviewees did not elaborate on whether this was due to the inconvenience of standing water in the community or because of the connection between standing water and permafrost degradation.

### Identifying the enabling and limiting factors for effective climate change adaptation in Arviat

This evaluation identified several enabling factors for effective climate change adaptation in Arviat, including institutional organization, public support for adaptation and stakeholder engagement. First, institutional organization, as defined by Ford and King (2015), considers how organizations coordinate and direct adaptation activities. This coordination is important for adaptation; it occurs where a government department, an interagency group, or a division within a department, takes a lead or coordinating role to reduce ad hoc adaptation. Our evaluation determined that the institutional organization of the ICCiLD project was strong. This network created greater synergy allowing project stakeholders to focus on their area of expertise. For example, in the ICCiLD project, cross-departmental coordination meant that:

- Government of Nunavut Community and Government Services used their technical development skills in framing the mapping project.
- The Department of Environment—Climate Change Section applied their knowledge of climate change engagement strategies.
- The Arviat Wellness Centre/Aqqiumavvik Society partnered locally to provide logistical support.

In the case of ICCiLD, this cross-scale institutional organization resulted in enhanced research communication and collaboration between different scales and stakeholder groups, including national university-level researchers, territorial planners and local service users:

The focus group that I attended was definitely the technical focus group, so it had the three levels of housing who were present, it had the mayor … the guys from Memorial University doing a presentation and the guys contracted to do the mapping doing a presentation and also [the Department of Environment] were doing a presentation. (Project creator).

This collaboration allowed for knowledge sharing and increased dialogue between decision makers from the local to the territorial level, an element previously described as lacking in northern hazard mapping research (Champalle et al. 2013).

Another enabling factor for climate adaptation was the high degree of public awareness for climate change. Public consciousness of climate change as a global phenomenon and its drivers was high in Arviat and the term ‘climate change’ was well-understood among interviewees. Public awareness of climate change is important for gaining buy-in and support for adaptation action. The project creators recognized the importance of raising awareness about local climate impacts in Arviat, and chose to increase community engagement during results dissemination, rather than just focusing on technical project end-users:

[They] produce the maps [and] work with the contractor … to put the maps in the community plans but we realized that the maps and the plans maybe weren’t being disseminated to the communities and it’s nice to get feedback (Project creator).

Through engaging with community stakeholders as well as technical users, the project raised the profile of permafrost degradation and the need for adaptation and planning for climate change in Arviat. Many interviewees recognized the need for adaptation:

I think changing is harder for us because we’re used to like the cold and things being constant but now things are getting more extreme and I guess we have to adapt to it. (Project user).

Despite the presence of enabling factors for adaptation readiness in Arviat, a significant barrier existed in the limited systems and methods for ensuring that ‘IQ’ was integrated into project outputs. IQ was collected through ICCiLD via informal meetings with community members during field visits and through more formalized meetings held with the Elders to discuss permafrost degradation. The IQ collected was collated into a summary document and is generally considered in community planning. However, there was no specific system in place for ensuring that the individual pieces of data would be included as layers in the hazard maps (see discussion for more on this).

Finally, interviewees identified decision-making as a barrier to adaptation readiness in Arviat. While decision makers were engaged in ICCiLD, interviewees stated that permafrost research in Arviat had some way to go in supporting climate conscious decision-making. Decision makers were looking to the research community for answers to other questions, including understanding the correct building foundation type to use in Arviat and the differential cost of adapting to climate change. Interviewees discussed an interest in research exploring the economic costs of adapting or not adapting to climate change. Interviewees believed climate change adaptation actions conflicted with immediate housing and infrastructure needs due to the additional cost of taking climate change into account. In a region with many pressing needs and limited financial and human resources, climate change was referred to by one interviewee as considering the: “sofa choice of a house whilst the foundations were still being built” (Project user). Interviewees identified a lack of consideration of other determinants affecting development decisions, particularly the cost of developing in certain areas and ownership of lots in communities:

I think land tenure issues have a huge significant impact [if a stakeholder already owns a plot of land] it’s no added cost for them in terms of land tenure … they can build on that piece of land already because there’s space, that already saves them $150–300,000, granted they might end up spending that much or more on maintenance in the future. (Project user).

## Discussion

Three overarching themes emerge from the evaluation interviews outlined above. First, objective and subjective risk perception; second, the need for coordination on planning for climate change and development; and third, Western and Inuit philosophies of planning and adaptation. These themes act as underlying challenges or opportunities in hazard mapping and are examined further in the discussion.

Risk as a concept is both objective and subjective. The objective form of risk considers “the potential physical harm to human beings, cultural artefacts or ecosystems and use(s) probabilities and expected values to express uncertainties and frequencies” (Aven and Renn 2010). The subjective form of risk is mentally constructed and based on personal beliefs, effects and experiences, which do not exist independent of its assessor (Aven and Renn 2010). Sutherland et al. (2012) introduce the concept of ‘riskscapes’ as a way to understand that individuals carry their own risk narratives which include past experiences with that risk and its perceived acceptability. Research on ‘riskscapes’ addresses a common issue in climate change research, a mismatch in perceived risk.

The interviews conducted for this evaluation suggest different perceptions on the challenge posed by permafrost degradation among scientists and the community. Risk governance in relation to the ICCiLD project must consider both aspects of risk, where the objective and calculated risk of surface subsidence are considered alongside individual community members’ perception of risk. The ICCiLD project outputs addressed key adaptation requests from decision makers in the territory. Creators expressed that Arviat was a strong candidate for the mapping project due to the limited local information available on permafrost conditions and because of the high community growth rate which necessitates the creation of tools and information for future development. At the regional (Eastern Canadian Arctic) level, hazard mapping for permafrost degradation was identified by representatives from Arctic communities and the territorial government as a priority for adaptation management (Black et al. 2012). Despite the perceived risk of permafrost thaw to infrastructure in Arviat at the regional and territorial level, disconnect between local development priorities and those addressed in the ICCiLD project was discernible in the interviews. The recommendations put forward by community interviewees, for instance, did not align with the hazard maps produced. Instead, recommendations were linked to alternative community priorities. For example, the Hamlet was concerned about the adverse impact of drainage issues on infrastructure and gave it a higher priority than permafrost degradation. In addition, interviewees talked about the quality of life being potentially constrained by adaptation and expressed statements in response to areas ranked as suitable on the map which included: not being located near the cemetery, the need to consider areas of polar bear activity, and a reluctance to be located close to the community dump. Individual ‘riskscapes’ have significant implications for the success of adaptation projects and while ICCiLD demonstrated some best practices in project design (e.g. increased community engagement and results dissemination), greater emphasis on risk perception is needed in future work.

Differences in risk perception underline the complexity of developing hazard maps as an adaptation. The adaptation process is more than a technical exercise of supplying information on landscape susceptibility to permafrost degradation. Hazard mapping exercises would benefit from improved coordination between all players. Preston et al. (2011) systematic review of vulnerability mapping advocates for the inclusion of more end-users during map creation. In the case of the ICCiLD project, the inclusion of housing staff at the project design stage could have created ground suitability categories which were linked to guidelines on the size of structures. Thus, providing applied adaptation advice, to increase utilization of map suitability categories in development decisions. Additionally, local coordination could have led to further on the ground validation of the area of disputed suitability ranking identified by interviewees during this evaluation. This may have reduced local distrust in the map outputs expressed by some interviewees.

In northern Canada, the complexity of these planning decisions is compounded by different worldviews and knowledge systems embodied in science and Inuit philosophies on planning (Bates 2007; Berkes 2009). Creating maps, which represent Western scientific methods, risks privileging Western science above Indigenous ways of knowing (Castleden et al. 2017). This prioritization of one knowledge system over another is problematic because Indigenous knowledge provides valuable historical and context-specific data in an area where limited Western scientific data exist. Additionally, Canadian Arctic governance structures also require the inclusion of this knowledge through the administration of the land claims agreement (NTI et al. 2010). Whilst technical data on permafrost thaw is important for adaptation decision-making, there is further work to be done on adequately incorporating different worldviews and philosophies when considering climate change adaptation to ensure that adaptation options fit with Indigenous philosophies (Tester and Irniq 2008). Whilst the ICCILD project did collect Indigenous knowledge relating to observations of landscape changes in the area over time, interviewees seemed unsure or uncertain as to how that information would be incorporated into the project. Interviewees also indicated that they thought the project design lacked significant consideration of how Indigenous philosophies and worldviews might impact project success. Similar concerns over the ability to incorporate and utilize Indigenous knowledge into adaptation planning decisions in Nunavut were also raised in Ford et al. (2017). Inuit planning philosophies are often based on an acceptance that the future will be uncertain, in which high value is placed on flexibility (Bates 2007). One of the four Inuit maligait (natural laws) describes the notion of continually planning/preparing for a better future, often linked to sustainability for future generations. In contrast, the Western perspective of trying to reduce uncertainty and predict future events is often seen as rigid, inflexible, and even arrogant (Bates 2007; Fienup-Riordan 2010). Work in Alaska on ‘Cultural Theories of Risk’ and climate change adaptation highlight worldviews, which are based on a respectful and ethical behaviour and the practice of remaining in balance with the land. The concept of permanent settlements can somewhat contradict with these Indigenous laws of sustainable land stewardship. This contrasting worldview on what is seen as sustainable planning may play a part in how well-received a climate change adaptation project is in a community and ultimately, whether the project outputs are adopted and used by community members. These worldviews can be at odds with state and federal systems, sometimes resulting in cultural and regulatory barriers to adaptation (McNeeley and Lazrus 2014; Tester and Irniq 2008). The literature suggests that the incorporation of Indigenous knowledge in adaptation planning is a challenge across northern regions and is not specific to this project alone (Chapin III et al. 2013; Ford et al. 2017; Knapp and Trainor 2013; Labbé et al. 2017). Table 2 provides suggestions on integrating Indigenous knowledge into a hazard map. Other potential tools for future hazard mapping research include the utilization of “clumsy solutions”, where discussions and workshops ensure multiple perceptions and values are captured in adaptation decisions (Verweij et al. 2014). Alternatively, fuzzy cognitive mapping techniques can also improve integration of different worldviews into hazard mapping projects (Özesmi and Özesmi 2004), allowing for representation of spiritual or cultural areas which should not be used for housing development and the incorporation of historical data on past community ground subsidence events.

**Table 2:** Barriers to adaptation and ability to overcome mapping barriers identified

Theme	Interview comments	Ability to overcome mapping barriers identified (strong, moderate, weak)
Local development preferences	“It would be safer if the houses were not spread apart and built only on green [suitable] land” (Project user) “Why would we want to be near the cemetery, our ancestors are buried there” (Project user)	*Moderate* The addition of points and lines on the map could highlight areas of contention and class those areas as unsuitable/marginal (e.g. Municipal dump and cemetery)
Integration of Indigenous knowledge	“What we thought wasn’t quite taken into consideration, knowing that, why would I trust it?” (Project user) “In IQ you have a way of viewing the world in a way that is totally holistic, in … western world view, you separate everything into its little silo and you deal with each one separately” (Project creator) “You need to combine these two, both knowledge’s together to fully see what’s what we’re seeing” (Project user)	Weak* Addition of points on the map such as historical knowledge on ground disturbance and class those areas as unsuitable/marginal *This is only one aspect of Indigenous knowledge (See discussion)
Cost of adaptation	“Because something is unsuitable or marginal we’re just not going to put anything there?” (Interview 06, Project user) “They shouldn’t be taking the route just because it’s cheaper, it should be the right one because there’s costs down the line” (Project creator) “I think you have to be kind of a wealthy society to be able to start planning ahead for things like climate change” (Project user)	*Moderate* Additional technical information for map suitability categories to improve ability for decision makers to determine the cost of building in unsuitable/suitable areas
Competing community priorities	“There is so much to do in Nunavut, there are so may needs … we’re struggling with education, we’re struggling with infrastructure, everything” (Project user)	*Weak* Difficult to integrate into a map format as often subjective

## Conclusion

Permafrost degradation in the Arctic is expected to accelerate with climate change, with implications for the sustainability of infrastructure, economic development, and traditional livelihood activities. Hazard maps are being used with increasing frequency as a tool for identifying hazardous areas and guiding community development across the north. Despite the increased utilization of hazard mapping, few studies have evaluated end-user perspectives on their effectiveness and usability in decision-making. This evaluation of a permafrost hazard mapping project in the community of Arviat, found that the ICCiLD project added new and relevant information for community planning, raised awareness of the local impacts of permafrost thaw, and improved stakeholder relations across scales. However, despite these advantages, interviewees reported that the maps created are not currently being utilized in community decision-making, reflecting ongoing data access challenges, a need to create technical guidelines for housing developers, and the existence of competing policy priorities. The maps will be integrated into planning and development documents for Arviat, which will influence development planning over the next 20 years in the community. This is an essential step in ensuring climate adaptive planning, and future work is planned to provide an outcome-based evaluation to explore the degree to which the community hazard map was utilized in the planning process.

In addition to these context-specific findings, this paper also identified some best practices for future hazard mapping projects across Arctic communities (see Text Box 1). The evaluation illustrates that a combination of community engagement alongside map production can address several critiques of previous hazard mapping projects. The findings underpin the importance of engaging community members and users into adaptation projects, to improve local data access, knowledge sharing, the applicability for local decision-making, and coordination of resources and organizations.

Best practices for hazard mapping in Arctic communities **Consult with decision makers** Include decision makers from all levels as early as possible in the process. In research design, local decision makers have pertinent insight on community priorities.

**Zoom out** Broaden the conversation and allow for exploration of the complex linkages between climate change impacts and other key policy decisions.

**Coordinate research** Increase engagement with those working on similar issues to find synergies.

- Locally to minimize overlap and increase ability to cross check
- Regionally to standardize hazard maps to broaden understanding and applicability.
- Nationally to aid co-learning and improve funding efficiencies..

**Integrate different ways of knowing** Integrate Indigenous knowledge systems into the mapping outputs, to reduce distrust in the work, and to uphold Indigenous land claims agreements. If data cannot be integrated into the map, a summary document should be attached to all reports to avoid the two pieces of information being interpreted separately (i.e. without the full data set).

**Provide data access** Ensure easily accessible data and understandable outputs. Consider whether the provision of working documents throughout the process may also improve the timeliness of outputs for decision-making.

**Operationalize the work** Consult with decision makers to improve map usability and operationalize outputs. Tailor outputs to the local context. Considering what map categories mean for structural risk, foundation choices, or maintenance can improve usability of outputs.

## Electronic supplementary material

Below is the link to the electronic supplementary material.

Supplementary material 1 (DOCX 64 kb)

## Footnotes

1 Aboriginal Affairs and Northern Development Canada changed their name during the project to Indigenous and Northern Affairs Canada (INAC). Following this, in August 2017, a plan was announced to dissolve INAC and create two new government departments: Indigenous Services Canada and Crown-Indigenous Relations and Northern Affairs Canada.

2 RADARSAT interferograms are used to detect changes in the Earth’s surface. They show the difference between the return signal of two radar measurements recorded at separate times for the same point on the ground.

3 In Arviat, this classification would have deemed most of the community unsuitable for development because of the large number of local water bodies. Thus, categorization of risk for Arviat was altered to within 10 m of a water body.

4 A confidence level is used to quantify the likelihood that the data shown are true.

5 The ‘evaluators’ of this project (the authors) were not involved in the design stage of the ICCiLD project.
