# Supplementary material for: Evaluating the effectiveness of hazard mapping as climate change adaptation for community planning in degrading permafrost terrain
Source: Sustain Sci. 2018 Aug 4;14(4):1041–56. doi: 10.1007/s11625-018-0614-x (PMC6563524; doi:10.1007/s11625-018-0614-x)
Supplement: Supplementary file 1 — Supplementary material 1 (DOCX 64 kb) [file 11625_2018_614_MOESM1_ESM.docx]

**Electronic Supplementary Material_** Evaluating the effectiveness of hazard mapping as climate change adaptation for community planning in degrading permafrost terrain.

Melanie Flynn¹, James Ford¹, Jolène Labbé², Lothar Schrott³, Shirley Tagalik⁴

¹ Priestley International Centre for Climate, University of Leeds *Leeds, UK*

² McGill University *Montreal, Canada*

³ United Nations University – Environmental Risk and Human Security and University of Bonn *Bonn, Germany*

⁴ The Arviat Wellness Centre/Aqqiumavvik Society *Arviat, Canada*

For all correspondence: gy08mjf@leeds.ac.uk

Optional Link

**Fig. 1** Evaluation framework describing the main components of the evaluation methodology

**Fig. 2** Logic model for Incorporating Climate Change into Land Development – Terrain Analysis project. Source: Authors own. Created using information from project summaries, Funding proposals and Annual reports. Abbreviations: AANDC (Aboriginal Affairs and Northern Development Canada), CGS (community and Government Services), DOE-CCS (Department of Environment – Climate Change Section), GN (Government of Nunavut)

Table 1 Interviewee categories used for evaluation of the project

| Interview group | Definition | Stakeholders |
| --- | --- | --- |
| Creators (Technical) | Those designing the technical monitoring parts of the hazard mapping project. It includes the government department who designed the project, those involved in the ground truthing of data and in-situ data collection. | Community and Government Services  3vGeomatics  University students |
| Creators (Community outreach) | Those responsible for the deliverables for the community engagement part of the project, including those organising activities for the community. | The Arviat Wellness Centre/ Aqqiumavvik Society  The Climate Change Section (Government of Nunavut) |
| Users (Technical) | Those expected to use the hazard maps and other technical outputs of the project for community planning purposes. | Community and Government Services  Nunavut Housing Corporation  Arviat Housing Association |
| Users (Community outreach) | Those community members who were engaged in either of the projects as an end user or as someone who will be affected by the community plans which will be created. | Elders  Local youth  Local business owners |
| Users (Mixed) | Those who do not fall clearly into either category and are likely to use both the technical and the outreach sections of the research. | The Hamlet of Arviat |

Table 2 Overview of top three comments made in semi structured interview divided by stakeholder groups and by (+) = Positive comments (-) = Negative comments (REC) = Recommendations. N=Total number of comments. n=Number of comments within that category

| Creator comments (+) (N=102) | User comments (+) (N=116) | |
| --- | --- | --- |
| Considered local context (n=16) | Increased knowledge sharing (n=22) |  |
| Aided in building relationships (n=16) | Local agreement with map (n=22) |  |
| Increased results dissemination (n=11) | Aids decision making (n=18) |  |
| Creator comments (-) (N=63) | User comments (-) (N=80) | |
| Lack of communication between project stakeholders (n=17) | Local knowledge contradicts data (n=13) | |
| Limited data access (n=6) | Limited data access (n=8) | |
| Timeliness of information (n=5) | Unclear ranking system on maps (n=5) | |
| Creator comments (REC)* (N=46) | User comments (REC) (N=45) | |
| Include more oral/engaging activities (n=6) | Don't build near water (n=5) | |
|  | Consider local quality of life (n=4) | |
|  | Clarification of ranking system on maps (n=4) | |
